# Association between subjective olfactory dysfunction and female hormone-related factors in South Korea

**DOI:** 10.1038/s41598-019-56565-x

**Published:** 2019-12-27

**Authors:** Kijeong Lee, In Hak Choi, Sang Hag Lee, Tae Hoon Kim

**Affiliations:** Department of Otorhinolaryngology-Head & Neck Surgery, College of Medicine, Korea University, Seoul, Korea

**Keywords:** Neurological disorders, Epidemiology, Risk factors

## Abstract

An association between olfactory dysfunction and female hormone level has been reported; however, no previous studies have investigated the correlation with life-long female hormone exposure. The aim of this study was to estimate the association between subjective olfactory dysfunction and various endogenous and exogenous female hormone-related factors including age at menarche and menopause, number of pregnancies and deliveries, age at first and last delivery, duration of breastfeeding, use of oral contraceptives, and use of hormone therapy. The study analysed a total of 3863 female participants using data from the Korean National Health and Nutrition Examination Survey V (2010–2012). The prevalence of olfactory dysfunction was 3.5% for premenopausal participants and 6.2% for postmenopausal women. Among premenopausal women (compared to women breastfed less than 12 months), the 12–24-month group (OR = 4.690, 95% CI = 1.431–15.369) and the 25–48-month group (OR = 6.548, 95% CI = 1.758–24.394) had higher rates of olfactory dysfunction. In postmenopausal women, starting menopause at a younger age was positively associated with olfactory dysfunction (OR = 0.939, 95% CI = 0.887–0.993). These data suggest that a longer duration of endogenous oestrogen deprivation is associated with subjective olfactory dysfunction.

## Introduction

Olfaction is a critical sensory function that is closely related to the quality of life^1^. Although the loss of olfactory function is not considered life-threatening, it decreases appetite and impairs the defence mechanisms against hazardous materials; this causes problems related to various aspects such as nutrition and emotion, which contribute to mortality^2–4^. The prevalence of olfactory dysfunction has increased due to a growing population of older adults and the increased prevalence of sinonasal diseases caused by pollutants. Olfactory impairment can be induced by various aetiologies other than ageing and sinonasal diseases, such as upper respiratory infection (URI), head trauma, and toxin exposure^5^. The prevalence of olfactory impairment is known to be higher among males, and it is thought to increase with age based on results from various population-based studies^6–8^. Nevertheless, post-viral olfactory impairment occurs predominantly in women between the fourth and sixth decade of life^5,9–11^. While no apparent explanation has been identified to date, female hormones are thought to be one of the factors that may be responsible for this phenomenon.

Several studies have reported an association between olfactory function and female hormones. A study of the olfactory performance of 332 women according to phases of the menstrual cycle reported that the odour detection threshold significantly decreased around the ovulatory phase^12^. Landis *et al*. reported that women who consumed oral contraceptives (OC) had better smell identification compared with those who did not, and a study of 432 postmenopausal women revealed that hormone replacement therapy improved odour memory as well as cognitive function^13,14^.

However, none of these earlier studies investigated the association between a personal history of life-long oestrogen exposure and olfactory impairment. This study aimed to determine the prevalence of self-reported olfactory impairment concerning endogenous and exogenous female hormone-related variables in premenopausal and postmenopausal women, respectively, based on data from a nationwide survey in South Korea.

## Results

### Baseline characteristics

Among the 3863 participants, 181 (4.0%) belonged to the olfactory dysfunction group (Table 1). The mean age of the olfactory dysfunction group (54.35 ± 1.222) was older than that of the control group (41.97 ± 0.359) (p < 0.001). Compared to the control group, more participants in the olfactory dysfunction group had a household income below the twenty-fifth-percentile (23.5%) (p = 0.003) and a level less than an elementary school education (34.9%) (p = 0.030). The distribution of residence location and occupation was similar between the two groups. In addition, no significant difference was identified in smoking habits between the groups (p = 0.633). In contrast, members of the olfactory dysfunction group consumed alcohol less frequently compared with the control group (p = 0.007). As for sinonasal diseases, only CRS showed a significantly higher prevalence in the olfactory dysfunction group (24.2%) than in the control group (4.0%) (p < 0.001).

**Table 1:** Baseline Characteristics.

Parameter	Olfactory dysfunction	Control	p-value
	n = 181 (N = 919 098)	n = 3682 (N = 20 485 394)	
Age	54.35 ± 1.222	41.97 ± 0.359	<0.001
Residence			0.143
Urban	135 (75.8)	3007 (81.4)	
Rural	46 (24.2)	675 (18.6)	
Household income			0.003
<25%	42 (23.5)	593 (15.4)	
25–50%	46 (31.3)	945 (28.2)	
51–75%	38 (15.6)	1053 (29.8)	
>75%	51 (29.6)	1039 (29.6)	
Education			0.030
Less than elementary	72 (34.9)	1127 (25.7)	
Less than high school	25 (16.5)	413 (12.3)	
Less than college	43 (30.4)	1164 (36.6)	
More than college	34 (18.1)	904 (25.5)	
Alcohol			0.007
Less than once a month	124 (71.1)	2003 (57.7)	
More than once a month	49 (28.9)	1271 (42.3)	
Smoking			0.633
Never/Ex-smoker	165 (94.9)	3073 (93.7)	
Current smoker	7 (5.1)	165 (6.3)	
Occupation			0.138
Administration	7 (5.4)	321 (10.1)	
Clerical work	3 (2.6)	198 (6.7)	
Sales/Service	26 (20.4)	462 (15.5)	
Agriculture/Fishery/Forestry	19 (7.7)	170 (3.9)	
Manual labour/Engineering	6 (4.0)	95 (3.0)	
Technical work/Assembling	15 (8.7)	314 (10.3)	
Homemaker/Student	96 (51.2)	1766 (50.5)	
Sinonasal disease			
Allergic rhinitis	39 (23.7)	557 (16.0)	0.082
Nasal septal deviation	76 (37.6)	1409 (42.2)	0.363
Chronic rhinosinusitis	46 (24.2)	125 (4.0)	<0.001

Data are presented as sample numbers (weighted percentages), and plus-minus values are means ± standard errors unless otherwise indicated, n: unweighted sample number, N: weighted sample number.

### Factors associated with olfactory dysfunction in premenopausal women

Among the 2183 premenopausal participants, 181 (3.5%) reported having olfactory dysfunction. Univariate logistic regression analysis showed that the duration of breastfeeding is significantly associated with the prevalence of olfactory dysfunction (Table 2). After adjusting for confounding factors, duration of breastfeeding showed a positive correlation with olfactory dysfunction in Model 1 (OR = 1.024, p = 0.016), Model 2 (OR = 1.030, p = 0.008), and Model 3 (OR = 1.036, p = 0.009) (Table 3). Further analysis was conducted by dividing the duration of breastfeeding into four groups. When compared with the participants who had been breastfeeding for less than 12 months, the 12–24-month group (OR = 4.690, p = 0.011) and the 25–48-month group (OR = 6.548, p = 0.005) had a significantly higher correlation with olfactory dysfunction in Model 3 (Table 4).

**Table 2:** Odds ratios for the association between female hormone-related factors and prevalence of olfactory dysfunction in premenopausal women.

	Mean ± SE		Unadjusted	
	Olfactory dysfunction	Control	Odds ratio (95% CI)	p-value
	n = 77 (N = 411 424)	n = 2106 (N = 12 583 196)		
Age at menarche	13.43 ± 0.261	13.49 ± 0.047	0.981 (0.824,1.167)	0.09
Number of pregnancies	2.94 ± 0.209	2.90 ± 0.045	1.017 (0.814,1.271)	0.880
Number of deliveries	1.51 ± 0.204	1.29 ± 0.038	1.220 (0.842,1.767)	0.820
Age at first delivery	25.65 ± 0.456	25.93 ± 0.127	0.980 (0.915,1.049)	0.557
Age at last delivery	29.40 ± 0.406	29.49 ± 0.134	0.995 (0.941,1.051)	0.847
Duration of breastfeeding (months)	10.38 ± 1.720	7.10 ± 0.288	1.018 (1.003,1.033)	0.020
Use of oral contraceptives, n (%)	10 (14.6)	57 (7.2)	2.086 (0.834,5.219)	0.116

n: unweighted sample number, N: weighted sample number.

**Table 3:** Multivariate logistic analysis in premenopausal women.

	Model 1		Model 2		Model 3	
	Odds ratio (95% CI)	p-value	Odds ratio (95% CI)	p-value	Odds ratio (95% CI)	p-value
Age at menarche	0.972 (0.745,1.267)	0.832	1.032 (0.787,1.353)	0.820	0.985 (0.720,1.346)	0.922
Number of pregnancies	0.892 (0.684,1.164)	0.400	0.903 (0.679,1.202)	0.485	0.850 (0.607,1.192)	0.345
Number of deliveries	1.078 (0.717,1.621)	0.718	0.957 (0.672,1.361)	0.806	0.853 (0.569,1.278)	0.439
Age at first delivery	1.127 (0.998,1.273)	0.054	1.120 (0.962,1.304)	0.145	1.115 (0.933,1.333)	0.230
Age at last delivery	0.909 (0.809,1.021)	0.108	0.907 (0.792,1.304)	0.159	0.897 (0.775,1.039)	0.146
Duration of breastfeeding (months)	1.024 (1.004,1.044)	0.016	1.030 (1.008,1.053)	0.008	1.036 (1.009,1.039)	0.009
Use of oral contraceptives	2.031 (0.651,6.334)	0.221	2.030 (0.743,5.550)	0.167	1.790 (0.549,5.832)	0.333

Model 1: adjusted for variables (p < 0.2) and age, Model 2: Model 1 + residence, household income, educational level, occupation, smoking status, and alcohol consumption, Model 3: Model 2 + allergic rhinitis, nasal septal deviation, and chronic rhinosinusitis.

**Table 4:** Multivariate logistic analysis according to breastfeeding duration in premenopausal women.

Duration of breastfeeding	Sample number		Model 3	
	Total	Olfactory dysfunction (%)	Odds ratio (95% CI)	p-value
<12 months	1676 (N = 2 923 220)	53 (N = 260 858) (2.5)	1 (reference)	
12–24 months	307 (N = 1 186 940)	13 (N = 99 096) (5.5)	4.690 (1.431,15.369)	0.011
25–48 months	179 (N = 528 877)	9 (N = 44 276) (4.5)	6.548 (1.758,24.394)	0.005
>48 months	21 (N = 66 017)	2 (N = 7193) (6.4)	7.069 (0.485,102.963)	0.152
P for trend				0.031

n: unweighted sample number, N: weighted sample number.

Model 3: adjusted for variables (p < 0.2), age, residence, household income, educational level, occupation, smoking status, alcohol consumption, allergic rhinitis, nasal septal deviation, and chronic rhinosinusitis.

### Factors associated with olfactory dysfunction in postmenopausal women

Among 1680 postmenopausal participants, 104 (6.2%) had impaired olfactory function. Univariate logistic regression analysis showed that only the age of delivering the first child showed a significant correlation with the prevalence of olfactory dysfunction (OR = 0.941, p = 0.035) (Table 5). After adjusting for confounding factors, the age at first delivery did not show statistical significance, and a younger age at menopause was significantly associated with a higher prevalence of olfactory dysfunction in Models 1–3 (Model 1: OR = 0.942, p = 0.017; Model 2: OR = 0.933, p = 0.009; Model 3: OR = 0.939, p = 0.028) (Table 6).

**Table 5:** Odds ratios for the association between female hormone-related factors and prevalence of olfactory dysfunction in postmenopausal women.

	Mean ± SE		Unadjusted	
	Olfactory dysfunction	Control	Odds ratio (95% CI)	p-value
	n = 104 (N = 507 674)	n = 1576 (N = 7 902 198)		
Age at menarche	16.10 ± 0.206	16.14 ± 0.043	0.991 (0.896,1.095)	0.856
Age at menopause	48.94 ± 0.428	49.70 ± 0.104	0.969 (0.932,1.007)	0.109
Number of pregnancies	4.79 ± 0.228	4.74 ± 0.051	1.011 (0.925,1.105)	0.809
Number of deliveries	3.27 ± 0.147	3.21 ± 0.038	1.022 (0.910,1.147)	0.714
Age at first delivery	24.29 ± 0.391	25.19 ± 0.103	0.941 (0.890,0.996)	0.035
Age at last delivery	30.55 ± 0.517	30.59 ± 0.115	0.998 (0.951,1.047)	0.930
Duration of breastfeeding (months)	55.26 ± 4.110	58.72 ± 1.095	0.998 (0.994,1.003)	0.475
Use of oral contraceptives, n (%)	26 (24.3)	330 (22.7)	1.089 (0.692,1.713)	0.713
Use of hormone therapy, n (%)	11 (10.3)	252 (14.1)	0.701 (0.391,1.324)	0.273

n: unweighted sample number, N: weighted sample number.

**Table 6:** Multivariate logistic analysis in postmenopausal women.

	Model 1		Model 2		Model 3	
	Odds ratio (95% CI)	p-value	Odds ratio (95% CI)	p-value	Odds ratio (95% CI)	p-value
Age at menarche	1.010 (0.895,1.140)	0.872	1.017 (0.899,1.151)	0.785	1.020 (0.894,1.165)	0.764
Age at menopause	0.942 (0.897,0.989)	0.017	0.933 (0.886,0.983)	0.009	0.939 (0.887,0.993)	0.028
Number of pregnancies	1.033 (0.893,1.193)	0.665	1.015 (0.874,1.177)	0.849	1.014 (0.867,1.187)	0.860
Number of deliveries	1.023 (0.802,1.304)	0.855	1.056 (0.823,1.353)	0.669	0.996 (0.764,1.298)	0.977
Age at first delivery	1.026 (0.953,1.105)	0.492	1.050 (0.969,1.137)	0.232	1.073 (0.987,1.166)	0.098
Age at last delivery	0.966 (0.912,1.024)	0.245	0.955 (0.899,1.014)	0.135	0.942 (0.883,1.005)	0.069
Duration of breastfeeding (months)	0.995 (0.987,1.003)	0.213	0.994 (0.986,1.002)	0.156	0.997 (0.988,1.005)	0.437
Use of oral contraceptives	1.114 (0.643,1.929)	0.701	1.165 (0.667,2.037)	0.591	1.288 (0.711,2.334)	0.404
Use of hormone therapy	0.576 (0.256,1.300)	0.184	0.508 (0.221,1.166)	0.110	0.493 (0.208,1.166)	0.107

Model 1: adjusted for variables (p < 0.2) and age, Model 2: Model 1 + residence, household income, educational level, occupation, smoking status, and alcohol consumption, Model 3: Model 2 + allergic rhinitis, nasal septal deviation, and chronic rhinosinusitis.

## Discussion

In the present study, we sought to investigate whether factors associated with exposure to female hormones were correlated with olfactory dysfunction in premenopausal and postmenopausal women, which has not been studied to date, as far as we know. The main findings of this study: (1) in premenopausal women, a longer duration of breastfeeding was positively associated with the prevalence of olfactory dysfunction, and (2) younger age at menopause showed a significant correlation with olfactory dysfunction in postmenopausal women.

The overall weighted prevalence of self-reported olfactory impairment based in this study was 4.0% of the adult females in this study. According to previous population-based studies, diagnosis of olfactory dysfunction based on self-reported subjective symptoms could underestimate the actual prevalence because subjects might be unaware of the impaired olfactory function depending on the degree of the symptoms and their age and race^15,16^. However, since most patients visiting the otorhinolaryngology department for olfactory alteration experience subjective discomfort, assessment of olfactory dysfunction based on a self-reported questionnaire could be meaningful. In addition, it is accepted that population-based studies based on self-report questionnaires could help establish groups that especially need medical attention or screening for specific diseases^17^. Thus, despite the limitation that the evaluation of olfactory performance was based on subjective symptoms, this study has importance because it suggests a possible aetiology for women who visit the clinic with olfactory deprivation and could contribute to establishing when subjects need early screening for olfactory performance before symptom present.

Previous studies that investigated sex differences with regard to olfactory perception or olfactory function alteration, based on female hormone fluctuations, reported conflicting results. Recently, Wang *et al*. reported that the gender effect on odour identification was observed only for young adults aged 18–50 years, based on a meta-analysis of 24 studies, suggesting the possible effect of oestrogen^18^. Furthermore, another meta-analysis study of 13 articles on the association between the menstrual cycle and olfactory sensitivity reported that olfactory thresholds were significantly lower during the fertile phase than during the non-fertile phase, indicating that olfactory function is the best when serum sexual hormone levels are at their peak^19^. However, none of the previous studies considered factors regarding overall exposure to female hormones throughout life. In our study, only the duration of breastfeeding had a significant association with olfactory dysfunction in premenopausal participants, and this association remained after adjusting confounding factors, including the presence of sinonasal diseases. The duration of breastfeeding can be the most prolonged period of female hormone deprivation during the reproductive period of a woman’s life, and this result indicates that a longer duration of oestrogen and progesterone deprivation during breastfeeding could be related to impaired olfactory function. Despite the absence of data about the weaning period (particularly, whether participants included the weaning period in their reported breastfeeding duration), we suggest that this does not have a significant impact on the outcome because the tendency of increased prevalence of olfactory dysfunction with a longer breastfeeding duration was prominent.

Regarding exogenous hormone use, history of OC use or maternal hormone therapy after menopause was investigated in this study. OCs (some of the most frequently prescribed medication types for women) regulate the natural female cycle through disruption of the hypothalamic-pituitary-ovarian axis by elevating the oestradiol concentration of the body; their impact on sensory perception or cognition have been reported^20,21^. Previous studies that suggested OCs affect the higher olfactory performance reported that the dose of oestradiol and duration of OC use are essential factors that influence the effect^22,23^. Further, another study of 33 women reported that the modulation of olfaction by OCs is limited to specific, odours, including aldosterone, androsterone, and musk^24^. Moreover, a prospective study that investigated changes in the olfactometric threshold during 17-β-oestradiol-drospirenone therapy revealed higher olfactory sensitivity during hormonal treatment than at baseline^25^. In this study’s results, neither OC use nor hormone therapy showed a significant correlation with olfactory dysfunction. However, this could be because detailed information of exogenous hormone use, such as the forms of hormone used or dose or duration of intake, could not be entirely ascertained from the survey.

In older populations, olfactory dysfunction is often related to cognitive impairment and neurodegenerative diseases such as Alzheimer’s disease or Parkinson’s disease, presenting with clinical symptoms preceded by neurologic symptoms. Deficiency of oestrogen has been considered a risk factor for cognitive dysfunction and neurodegenerative disease^26^. Recently, a study with data from 1315 women from a British-born cohort reported that delayed natural menopause was related to maintaining verbal memory. In contrast, age at surgical menopause or hormone replacement therapy was shown to have no significant effects^27^. Furthermore, in a study consisting of animal experiments, oestradiol deprivation by ovariectomy resulted in the degeneration of olfactory epithelium, which was restored by oestradiol replacement resulting in a thickened olfactory epithelium and increased numbers of matured olfactory neurons^28^. This study also suggests that the alteration of the olfactory system by oestradiol treatment is amplified by the presence of apolipoprotein E, a genetic risk factor for Alzheimer’s disease. These findings support the conclusion that a younger age at menopause is associated with olfactory dysfunction in postmenopausal women, suggesting that more prolonged exposure to endogenous female hormones could be associated with better olfactory function. These findings also suggest a relationship to maintained cognitive function, which was not estimated by the survey used in the present study.

The present study has limitations. First, because this study was a population-based cross-sectional study, the causal relationship between female hormone-related factors and outcomes is difficult to assess. Second, since variables related to female hormones were defined according to the patient self-reporting, recall bias could be present; in addition, it was difficult to distinguish between the effects of the two primary female hormones (oestrogen and progesterone). Furthermore, the present study could not determine the relative severity of olfactory dysfunction because KNHANES V did not include an olfactory function test or a detailed questionnaire. Despite these limitations, the strengths of this study are that it is the first to estimate a correlation between olfactory dysfunction and factors associated with life-long exposure to endogenous and exogenous female hormones. Further, the nationwide survey used in this study is representative of the whole population of Korea.

In conclusion, this nationwide population-based study indicates that a longer duration of endogenous female hormone deprivation is related to a higher risk of olfactory dysfunction. Before menopause, hormone deprivation due to breastfeeding was shown to be one of the most significant factors in this study; in postmenopausal women, age at menopause was an influential factor for olfactory dysfunction.

## Methods

### Data collection from the nationwide survey and study population

This cross-sectional study used data from the Korean National Health and Nutrition Examination Surveys V (KNHANES V) collected from 2010 to 2012. KNHANES is a nationwide population-based survey that is representative South Korean population outside of the industrial workforce; it includes a health interview, a nutritional survey, and physical examinations. The samples for the surveys were extracted by multistage-clustered probability sampling design based on National Census data. Among the 200 000 geographically defined primary sampling units (PSUs) in the entire country, 192 PSUs were randomly sampled, and 20 households in each of these PSUs were further selected. Health interviews and examinations were conducted at a mobile examination centre, and each field operation team consisted of 10 members, including trained medical staff, interviewers, radiologic technicians, and doctors. For the survey population to be representative of the entire South Korean population, the survey sample weights were constructed regarding the complex survey design, non-response rate, and a post-stratification that adjusted for of age and sex.

The Korean Society of Otorhinolaryngology-Head and Neck participated in the KNHANES V survey. Medical interviews and endoscopic examinations of the otolaryngology field were performed by 150 residents from the otorhinolaryngology departments of 47 institutes. Before conducting the survey, education for participating residents was conducted each year, the contents of which included the following: the overview of the survey; purpose of, and caution related to, the investigation of otolaryngology diseases; a detailed guidance on diagnosis and severity classification of diseases; and simulation training with the actual examination equipment.

The Institutional Review Board of the Korea Centers for Disease Control and Prevention approved this study protocol (2010-02CON-21-C, 2011-02CON-06-C, and 2012-01EXP-01-2C) according to the Ethical Principles for Medical Research Involving Human Subjects, as defined by the Helsinki Declaration. The enrolled populations provided informed consent before the survey.

**Figure 1:**
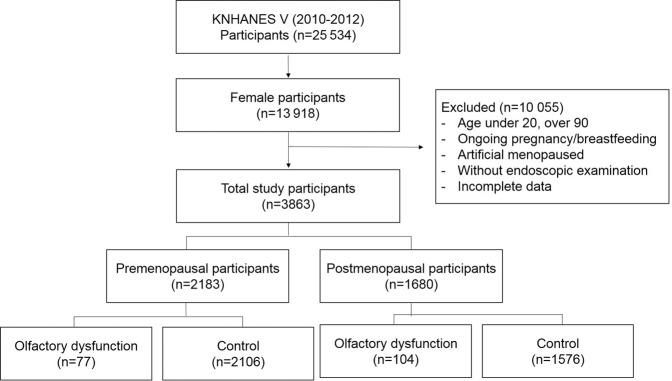
Flow chart used for patient selection, KNHANES: Korean National Health and Nutrition Examination Survey V.

Among the 25 534 participants who enrolled in the KNHANES V survey, 13 918 were females. Among these, the following participants were excluded—those under 20 years old and over 90 years old, pregnant or breastfeeding, undergoing artificial menopause, and who provided incomplete data. In addition, among premenopausal participants, participants over 65 years old were excluded based on a survey questionnaire, and participants under 35 years old were excluded from the postmenopausal subject group (Fig. 1).

### General characteristics

Baseline characteristics of the population (including age, residence, household income, education level, occupation, tobacco use, and alcohol consumption) were recorded. Residence was categorised as either urban or rural according to the participant’s official address. Household income was classified into four groups by quartiles: <25%, 25–50%, 51–75%, and ≥75%. Education level was categorised into four groups: less than elementary school, less than high school, some college, and a bachelor’s degree or higher. Participants’ occupation was classified by seven categories: administration, clerical work, sales-service, agriculture-fishery-forestry, manual labour-engineering, technical work-assembling, and homemaker-student. Participants were classified into two groups based on alcohol consumption (drinking four or more times a week or drinking less than four times a week) and tobacco use history (current and ex-smokers, or never a smoker).

### Assessment of variables

Olfactory dysfunction was defined from the answers provided to the question, “have you had problems with your sense of smell for more than three months or over the past twelve months?” The participants who provided an affirmative answer to this question were considered to have anosmia or hyposmia.

Participants who provided an affirmative answer to the question, “Have you ever been diagnosed with allergic rhinitis by a physician?” were given the diagnosis of allergic rhinitis. The nasal septal deviation was defined based on the endoscopic finding of asymmetrical deviation of the septum after shrinkage of the nasal mucosa. The presence of nasal polyps upon nasal endoscopic examination, or the presence of more than two of the following symptoms, resulted in the diagnosis of chronic rhinosinusitis (CRS): anterior or posterior nasal drip, nasal obstruction, facial pain or pressure, and olfactory dysfunction for at least 12 weeks based on the epidemiologic section of the European study on rhinosinusitis and nasal polyps (EPOS)^29^.

The questionnaire on female hormone-related factors included age at menarche, number of pregnancies, number of deliveries, age at first delivery, age at last delivery, duration of breastfeeding, and use of oral contraceptives. In postmenopausal participants, questions on age at menopause and use of hormone therapy were included.

### Statistical analysis

Statistical analyses of the data were performed with the complex sample module using SPSS version 20.0 (IBM Corp., Armonk, NY). KNHANES sampling weight variables were used to precisely estimate the general South Korean population based on the sample. A chi-squared test was used to evaluate and compare the relative weighted prevalence rates between the groups using the complex sample software module. Logistic regression analysis was performed to evaluate associations between olfactory dysfunction and female hormone-related factors. For the performance of multiple logistic regression analysis, variables with a p-value less than 0.2 from univariate logistic regression analysis were used. Three models calculated adjusted odds ratios (ORs) according to confounding factors: adjustment for variables with p-values less than 0.2 and age in Model 1; adjustment for variables with p-values less than 0.2, age, residence, household income, educational level, occupation, smoking status, and alcohol consumption in Model 2; and adjustment for variables with confounding factors in Model 2 and sinonasal disease including allergic rhinitis, nasal septal deviation, and chronic rhinosinusitis in Model 3. P-values less than 0.05 were considered statistically significant.

## Data Availability

The datasets generated during or analysed during the current study are available from the corresponding author on reasonable request.
